# Supplementary material for: An Expanded Lpt Pathway Couples Surface Lipoprotein Export to Outer Membrane Vesicle Biogenesis in Bacteroides
Source: bioRxiv. 2026 Oct 5:2026.10.02.756314. Preprint. [Version 1] doi: 10.64898/2026.10.02.756314 (PMC13692018; doi:10.64898/2026.10.02.756314)
Supplement: Supplementary file 1 [file NIHPP2026.10.02.756314V1-supplement-1.pdf]

bioRxiv preprint doi: https://doi.org/10.64898/2026.10.02.756314; this version posted October 5, 2026. The copyright holder for this preprint (which was not certified by peer review) is the author/funder, who has granted bioRxiv a license to display the preprint in perpetuity. It is made available under a CC-BY 4.0 International license.

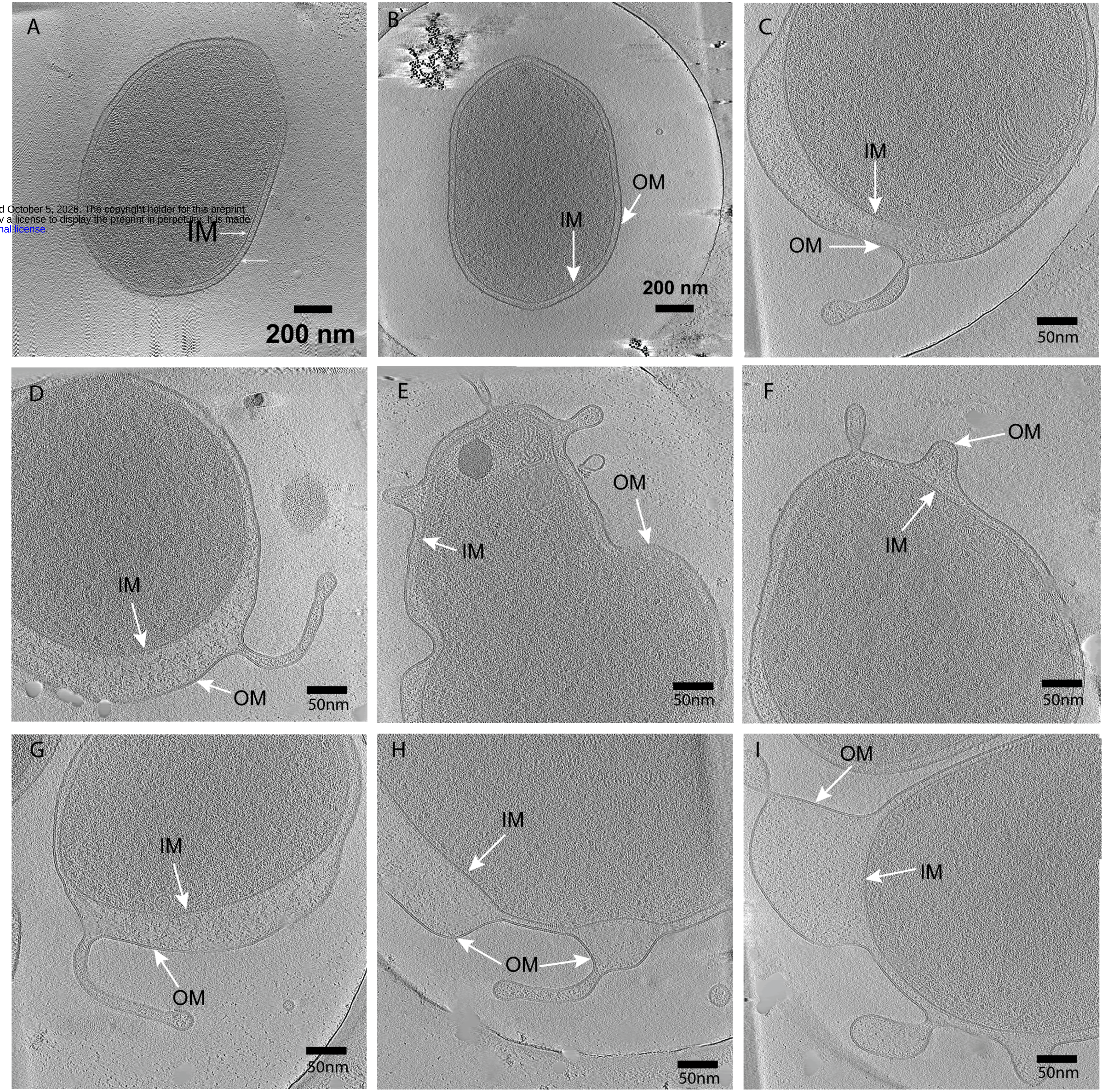

Figure S1: Slice through cryo-electron tomograms of WT (A-B) and Δ*lptZ* mutant cells (C-I) highlighting the irregular morphologies of mutant cells including extended periplasmic space and the presence of tubular structures.

bioRxiv preprint doi: https://doi.org/10.64898/2026.10.02.756314; this version posted October 5, 2026. The copyright holder for this preprint (which was not certified by peer review) is the author/funder, who has granted bioRxiv a license to display the preprint in perpetuity. It is made available under a CC-BY 4.0 International license.

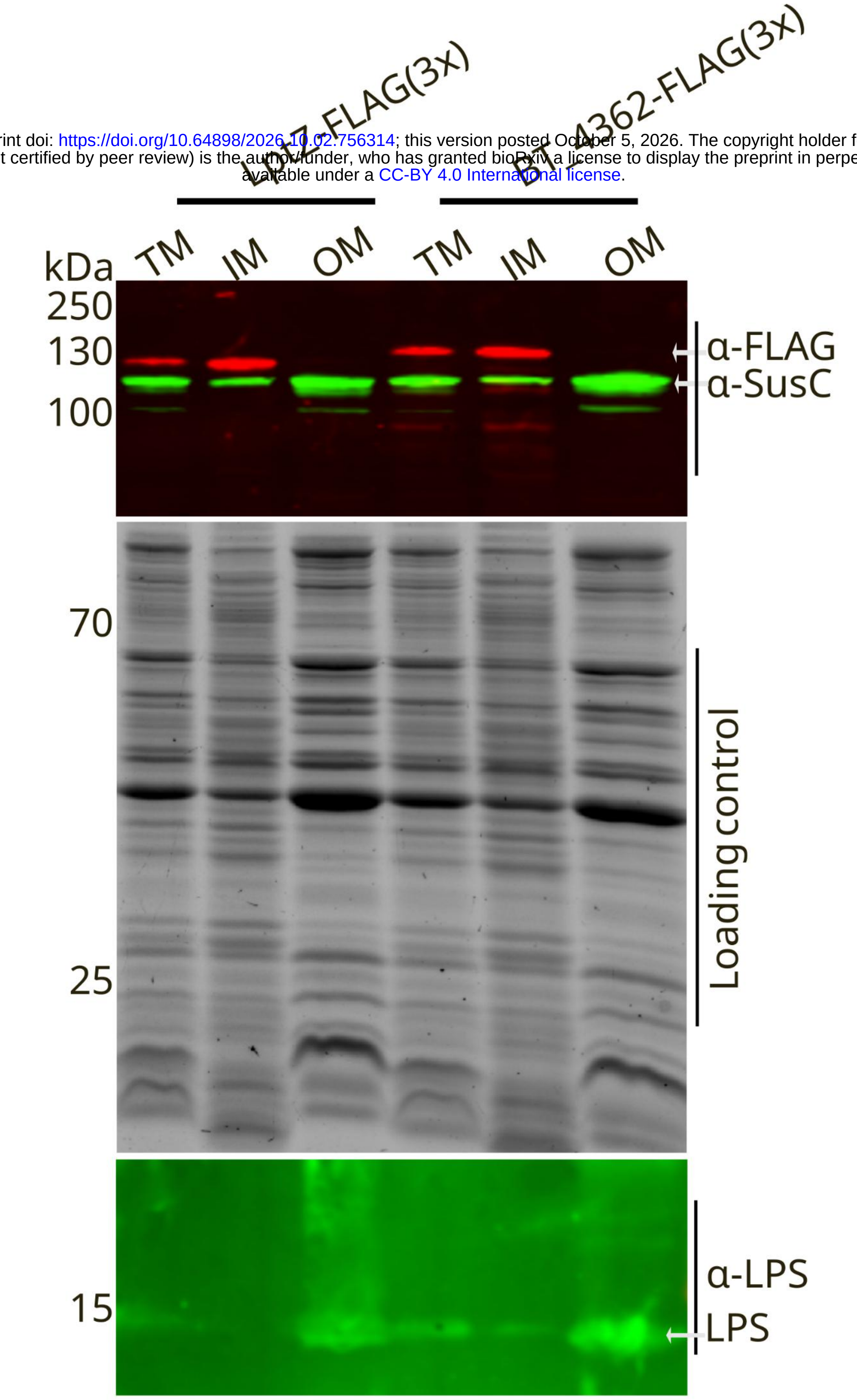

Figure S2: LptZ localizes to the IM. Upper panel. Western blot of FLAG-tagged LptZ and SecA (BT_4362) expressed in WT. LptZ shows IM localization predominantly. SusC (BT_3702) is found to be enriched in the OM fraction and serves as an OM marker. **Middle panel.** SDS-PAGE confirms equal loading. **Lower panel**. Western blot of proteinase K-treated TM, IM, and OM samples, probed with an anti-LPS antibody, reveals a specific LPS band (arrow) used as an OM control.

bioRxiv preprint doi: https://doi.org/10.64898/2026.10.02.756314; this version posted October 5, 2026. The copyright holder for this preprint (which was not certified by peer review) is the author/funder, who has granted bioRxiv a license to display the preprint in perpetuity. It is made available under a CC-BY 4.0 International license.

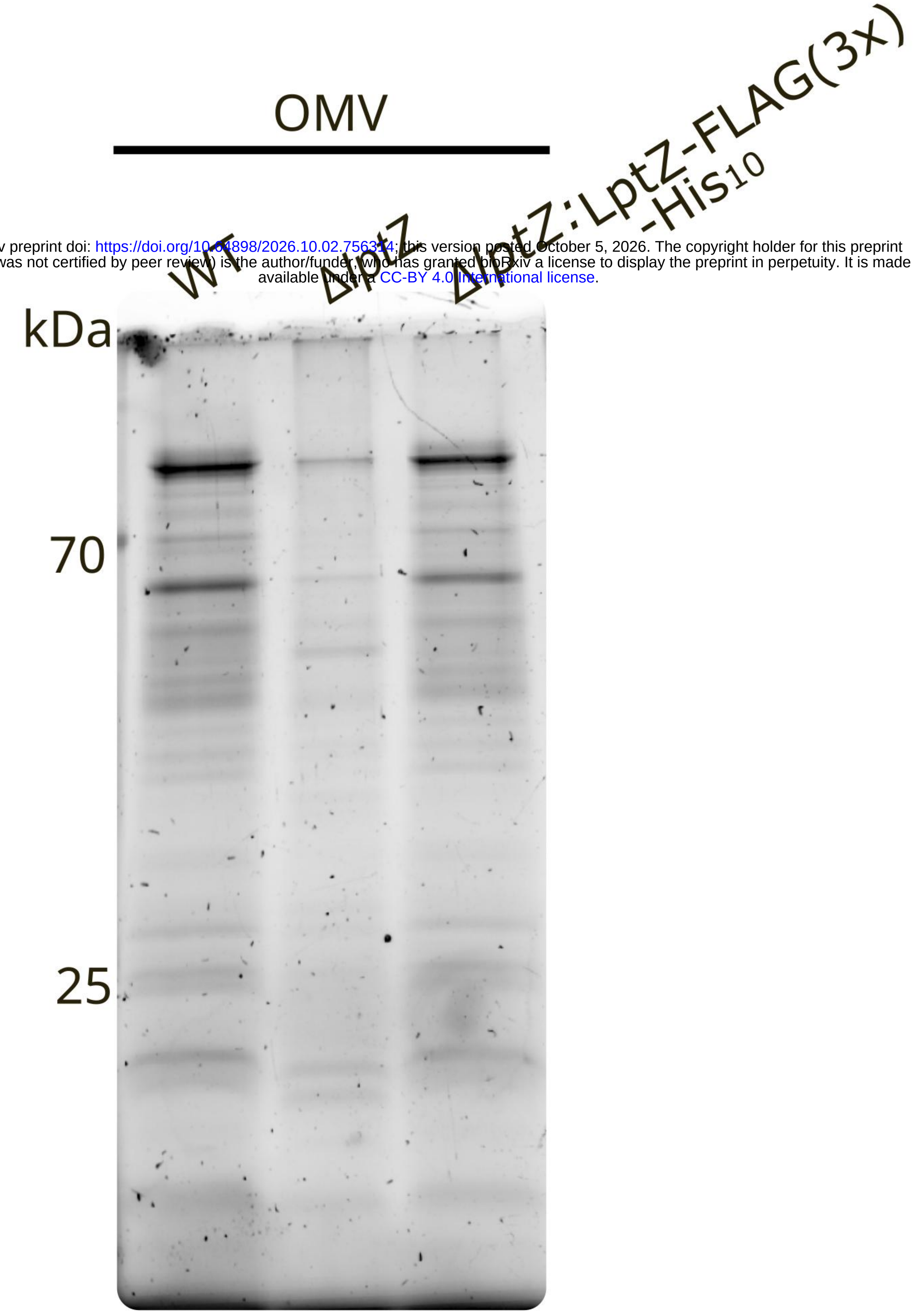

Figure S3: Dual-tagged (3xFLAG-His_{10}-tagged) LptZ retains its activity. OMVs were purified from WT, Δ*lptZ* mutant, and complemented (LptZ-FLAG(3x)-His10) strains and subjected to SDS-PAGE analysis. The similar OMV protein profiles of the WT and complemented strains indicate that the dual-tagging of LptZ does not affect its activity.

bioRxiv preprint doi: https://doi.org/10.64898/2026.10.02.756314; this version posted October 5, 2026. The copyright holder for this preprint (which was not certified by peer review) is the author/funder, who has granted bioRxiv a license to display the preprint in perpetuity. It is made available under a CC-BY 4.0 International license.

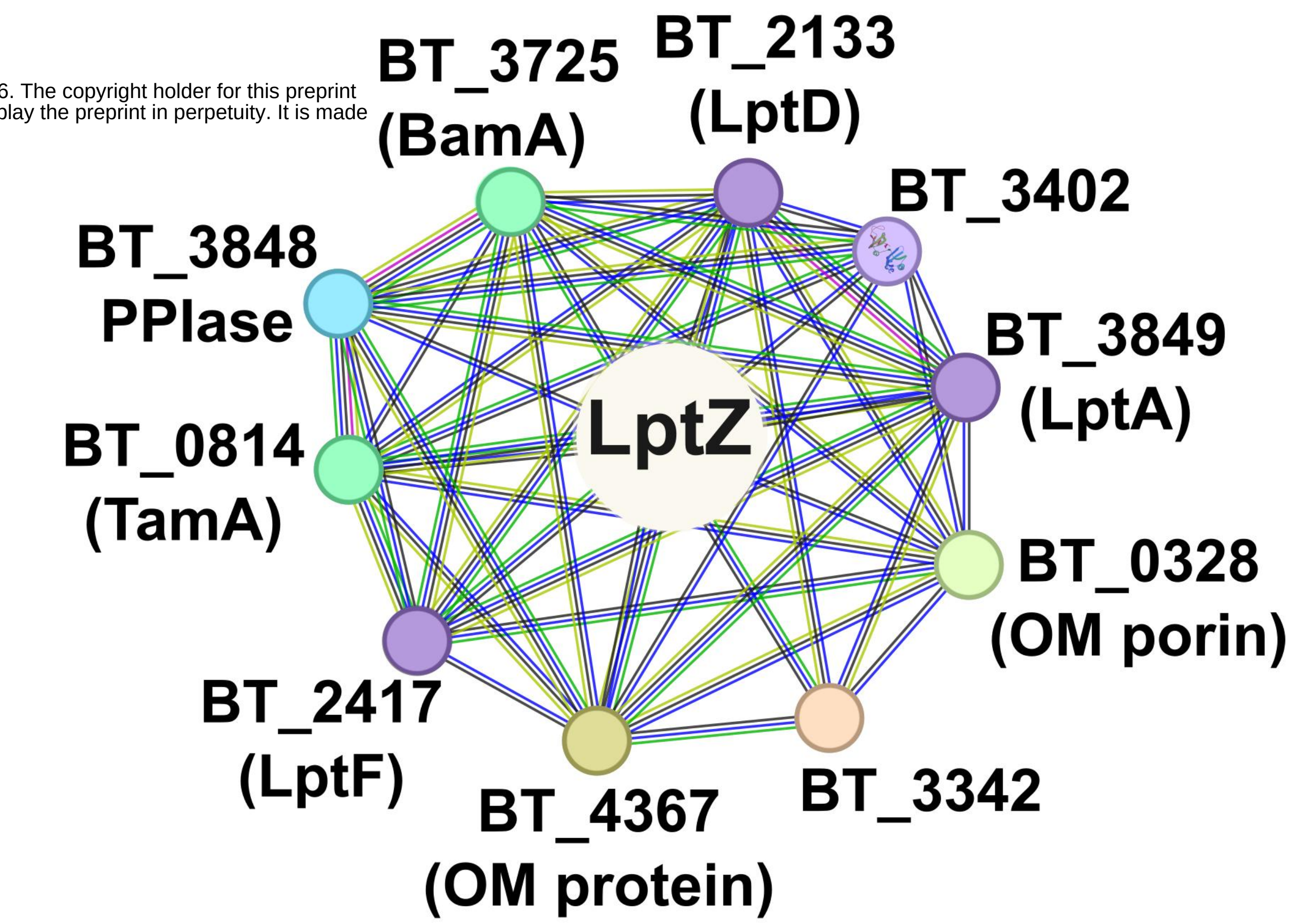

Figure S4 STRING-based interaction network of LptZ reveals predicted associations with LPS transport components (LptA [BT_3849], LptD [BT_2133], LptF [BT_2417]), β-barrel assembly factors (BamA [BT_3725], TamA [BT_0814]), and other OM-associated proteins (BT_3848, BT_0328, BT_4367). Lines indicate associations between proteins: Blue: known interactions from curated databases; Pink: experimentally determined; Green: gene neighborhood; Red: gene fusions; Light blue: text mining; Black: co-expression; Purple: protein homology.

bioRxiv preprint doi: https://doi.org/10.64898/2026.10.02.756314; this version posted October 5, 2026. The copyright holder for this preprint (which was not certified by peer review) is the author/funder, who has granted bioRxiv a license to display the preprint in perpetuity. It is made available under a CC-BY 4.0 International license.

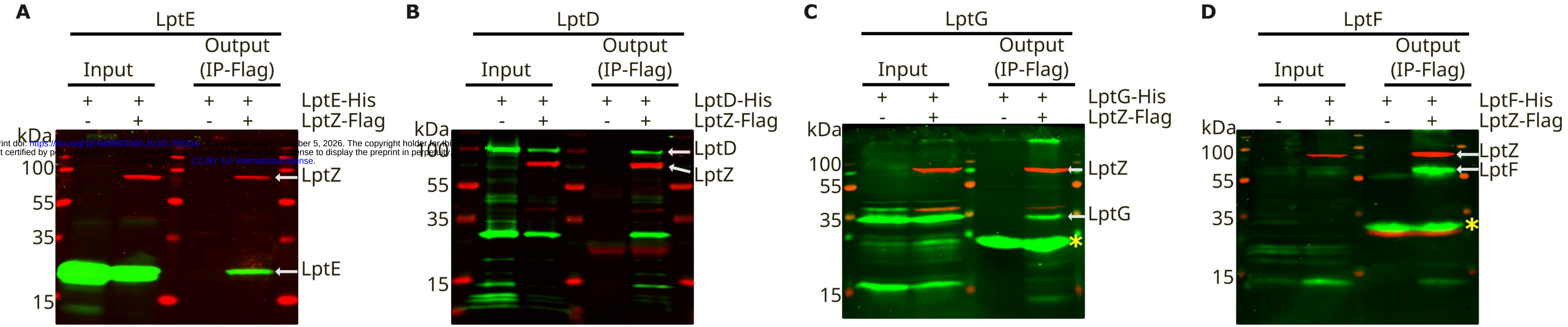

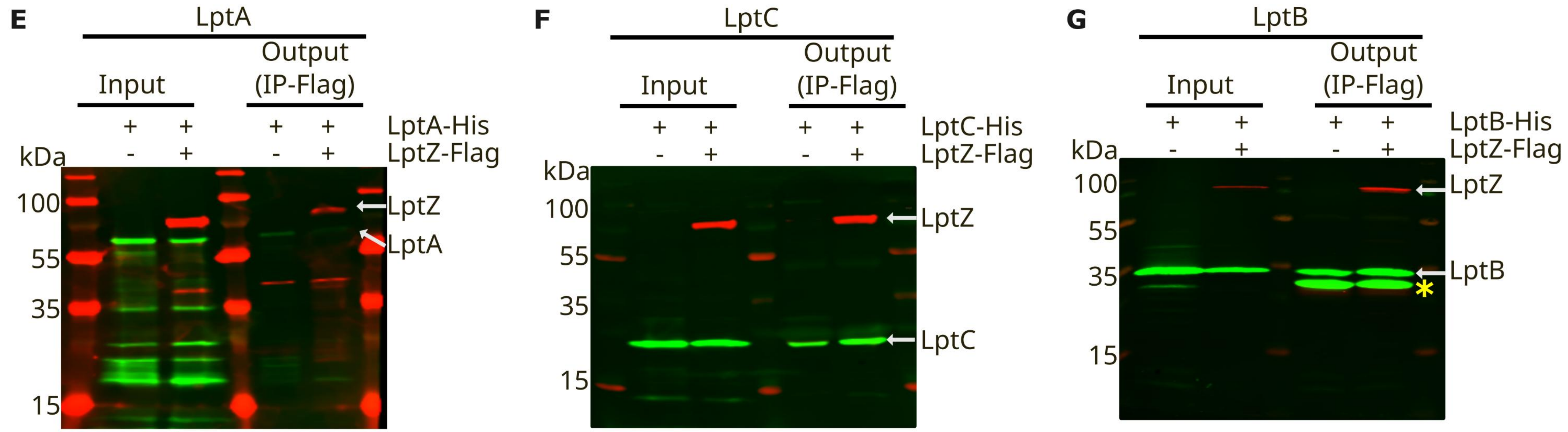

H

Lpt component	LptZ interaction	Lpt protein location
LptE	+	OM
LptD	+	OM
LptG	+	IM
LptF	+	IM
LptA	-	Periplasm
LptC	-	IM
LptB	-	IM

Figure S5: LptZ directly interacts with LptD, LptE, LptF and LptG. (A-G) LptZ selectively interacts with components of the Lpt transenvelope complex. **(A-G)** His-tagged LptA-G were co-expressed in *E. coli* with either empty vector (pBAD33.1) or FLAG-tagged LptZ (pBAD33.1:LptZ-FLAG). Lysates were subjected to anti-FLAG pulldown and immunoblotted with anti-His and anti-FLAG antibodies. Enrichment of His-tagged LptE (A), LptD (B), LptG (C), and LptF (D) in the presence of LptZ, compared to empty vector, indicates specific interactions. No enrichment of LptA (E), LptC (F), or LptB (G) was observed in the presence of LptZ compared to the empty vector, suggesting the lack of interaction with LptZ. Yellow stars indicate nonspecific bands from the anti-FLAG resin, which are absent in the input lanes. **(H)** Summary of the Co-IP analysis showing the Lpt proteins that co-purified with LptZ (+) and those for which no interaction was detected (-). Experiments were independently repeated at least three times with similar results; one representative result is shown.

bioRxiv preprint doi: https://doi.org/10.64898/2026.10.02.756314; this version posted October 5, 2026. The copyright holder for this preprint (which was not certified by peer review) is the author/funder, who has granted bioRxiv a license to display the preprint in perpetuity. It is made available under a CC-BY 4.0 International license.

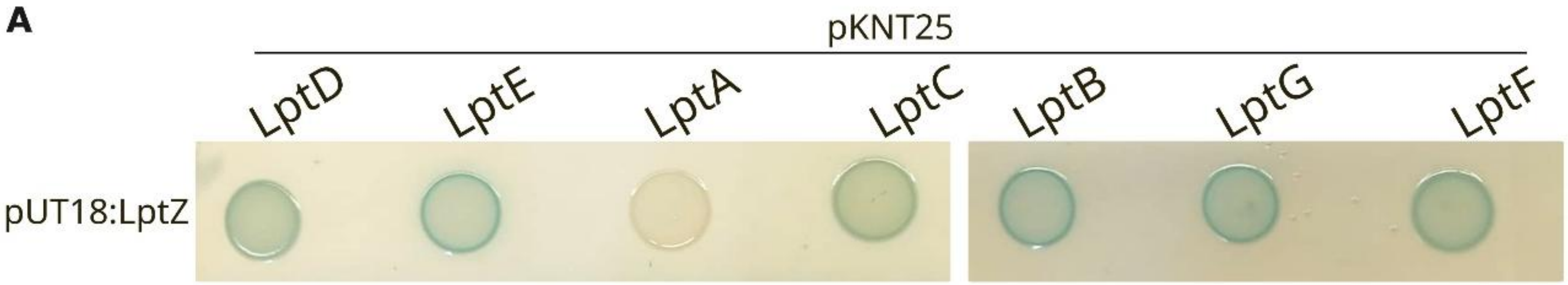

Figure S6. Bacterial two-hybrid assay to assess protein-protein interactions. Putative candidate proteins were fused to the T25 domain, and LptZ was fused to the T18 domain of *Bordetella pertussis* adenylate cyclase. Plasmids encoding the T18- and T25-fusion constructs were co-transformed into the *E. coli* BTH101 reporter strain. Protein-protein interactions were detected by spotting transformants onto LB agar plates supplemented with X-Gal and IPTG, and blue colonies indicate positive protein-protein interactions. Experiments were independently repeated at least three times with similar results; one representative result is shown.

bioRxiv preprint doi: https://doi.org/10.64898/2026.10.02.756314; this version posted October 5, 2026. The copyright holder for this preprint (which was not certified by peer review) is the author/funder, who has granted bioRxiv a license to display the preprint in perpetuity. It is made available under a CC-BY 4.0 International license.

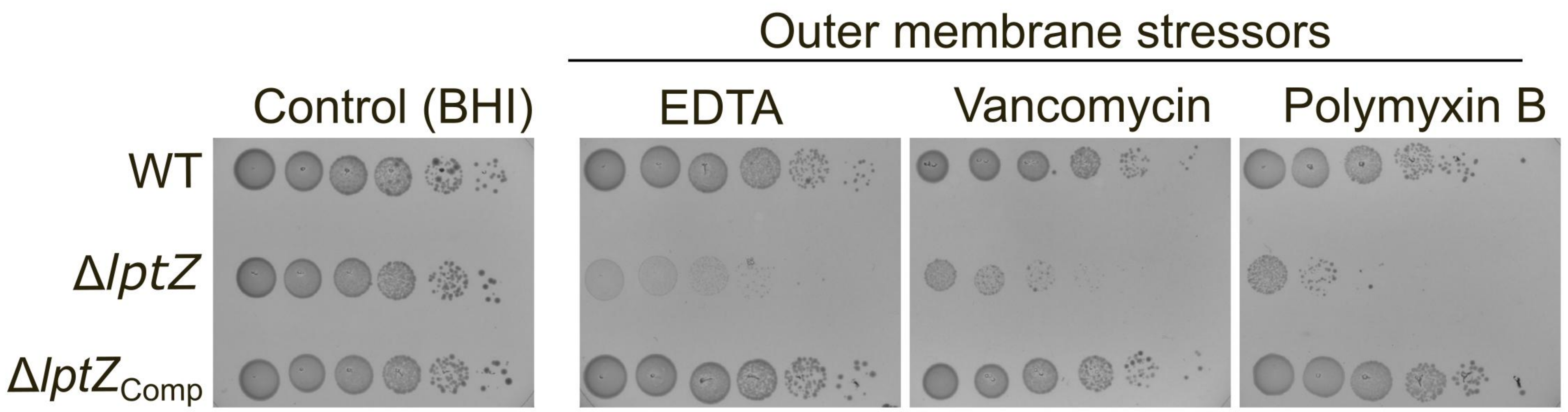

Figure S7. LptZ is required for outer-membrane integrity and resistance to envelope stress: Serial 10-fold dilutions of WT, Δ*lptZ* mutant, and complemented *Bt* strains were spotted on BHI agar with 1 mM EDTA, 16 µg/mL vancomycin, or 10 µg/mL polymyxin B. BHI alone served as a control. Plates were incubated anaerobically at 37 ºC for 48 h. Representative results from three independent experiments are shown here.

bioRxiv preprint doi: https://doi.org/10.64898/2026.10.02.756314; this version posted October 5, 2026. The copyright holder for this preprint (which was not certified by peer review) is the author/funder, who has granted bioRxiv a license to display the preprint in perpetuity. It is made available under a CC-BY 4.0 International license.

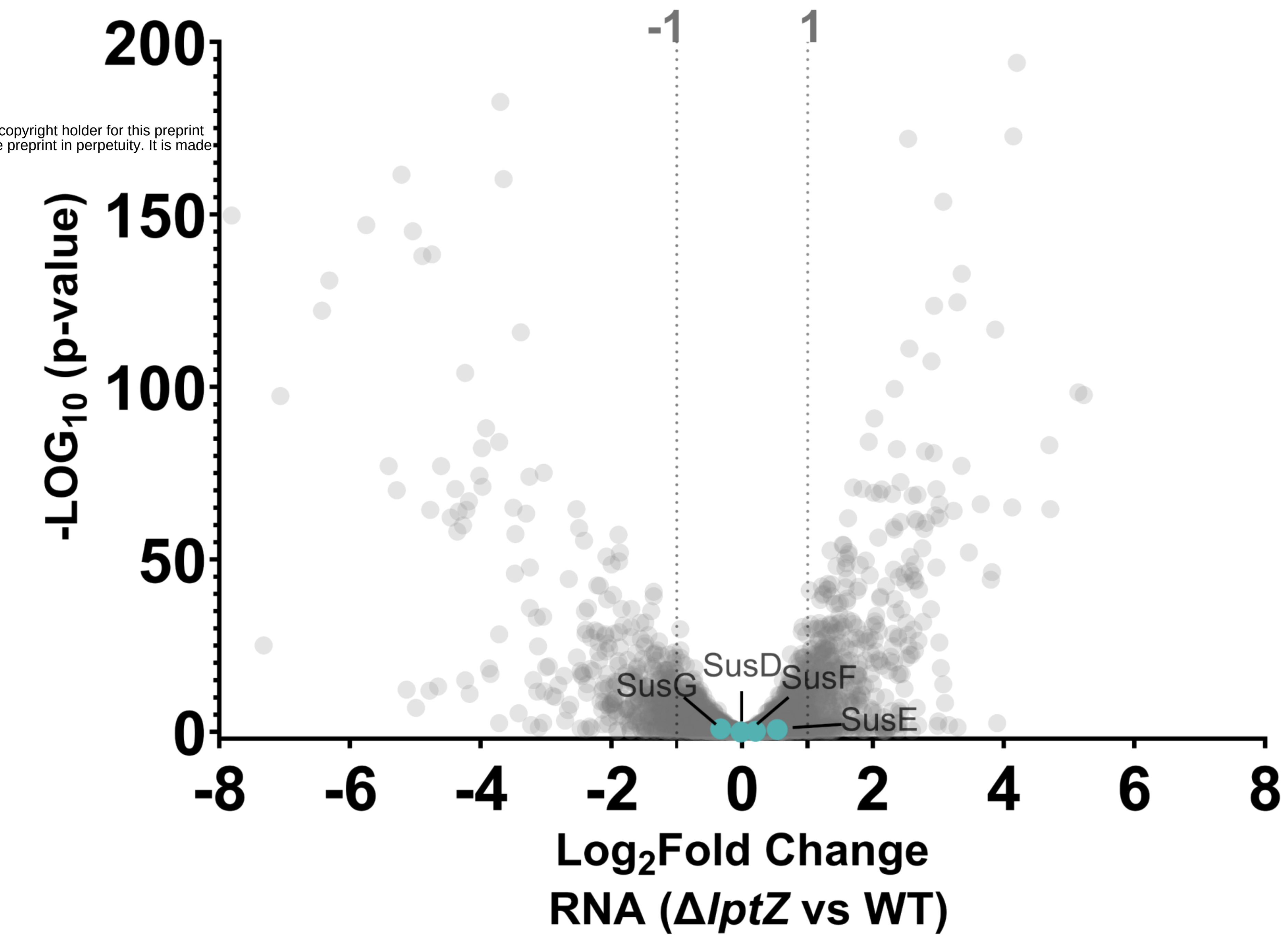

Figure S8: Volcano plot comparing transcript levels between WT and Δ*lptZ*. Dotted lines at $\log_2$(fold change) ±1 indicate unchanged expression. All *sus* genes fall within this range, showing no transcriptional change in Δ*lptZ*.

bioRxiv preprint doi: https://doi.org/10.64898/2026.10.02.756314; this version posted October 5, 2026. The copyright holder for this preprint (which was not certified by peer review) is the author/funder, who has granted bioRxiv a license to display the preprint in perpetuity. It is made available under a CC-BY 4.0 International license.

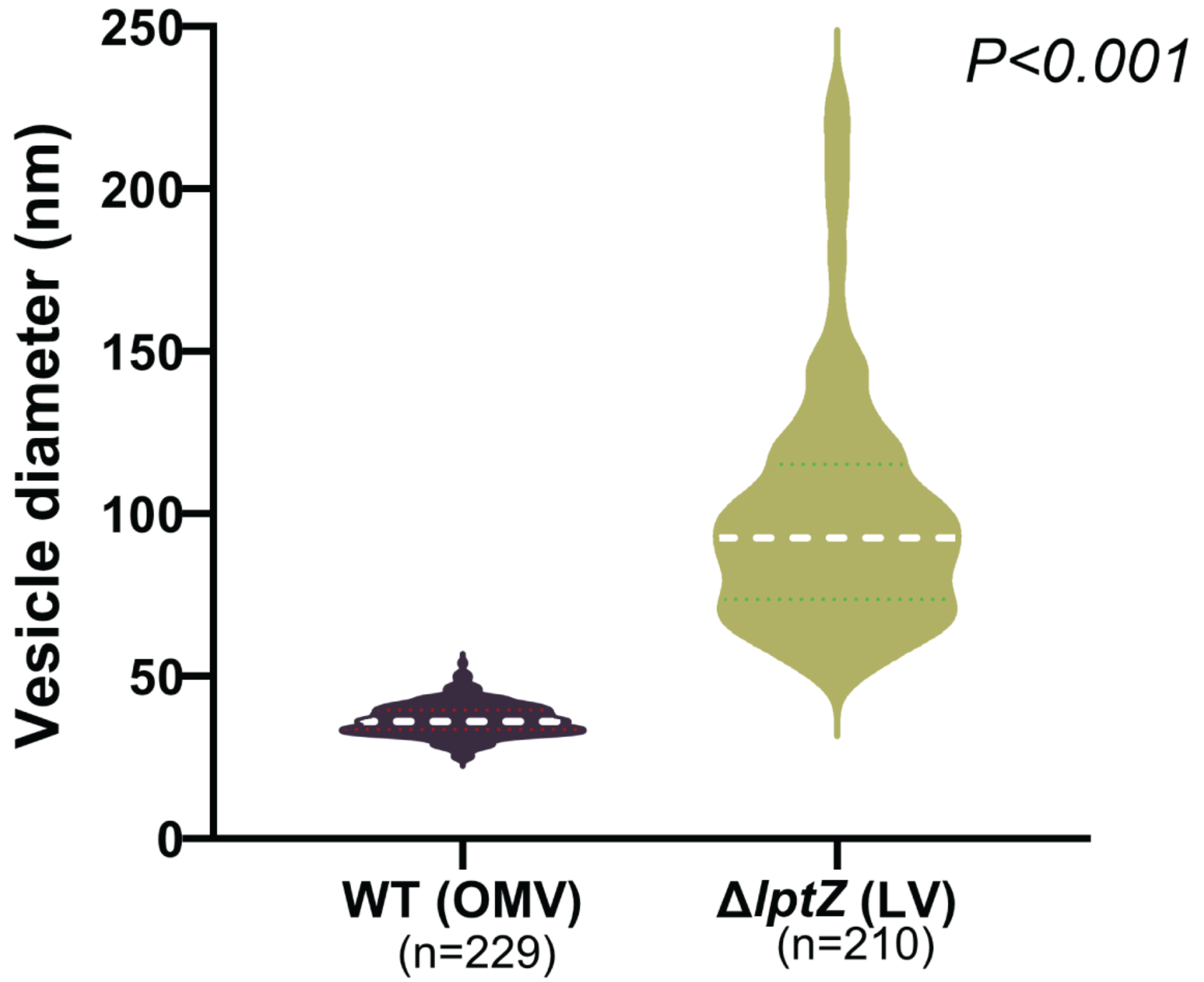

Figure S9. Vesicle diameters measured from cryo-ET images show a uniform size distribution in WT-derived OMVs, whereas LVs from the Δ*lptZ* mutant are highly heterogeneous and larger in size.

bioRxiv preprint doi: https://doi.org/10.64898/2026.10.02.756314; this version posted October 5, 2026. The copyright holder for this preprint (which was not certified by peer review) is the author/funder, who has granted bioRxiv a license to display the preprint in perpetuity. It is made available under a CC-BY 4.0 International license.

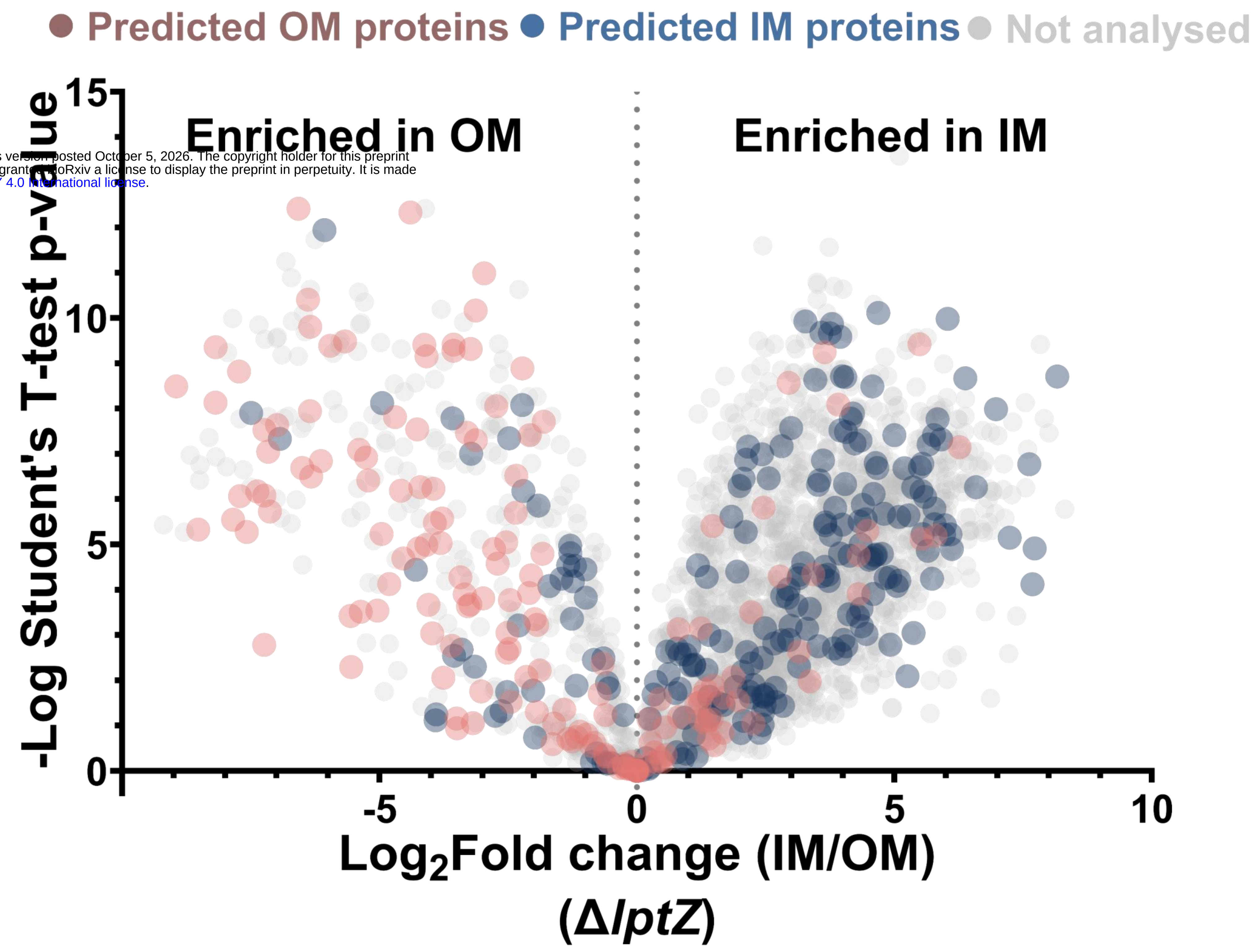

Figure S10: IM and OM separation in ΔLptZ. Volcano plot of isolated IM and OM proteomes of ΔLptZ, highlighting enrichment of predicted IM-specific proteins (blue circle) and OM markers (maroon circle) in their respective fractions.

bioRxiv preprint doi: https://doi.org/10.64898/2026.10.02.756314; this version posted October 5, 2026. The copyright holder for this preprint (which was not certified by peer review) is the author/funder, who has granted bioRxiv a license to display the preprint in perpetuity. It is made available under a CC-BY 4.0 International license.

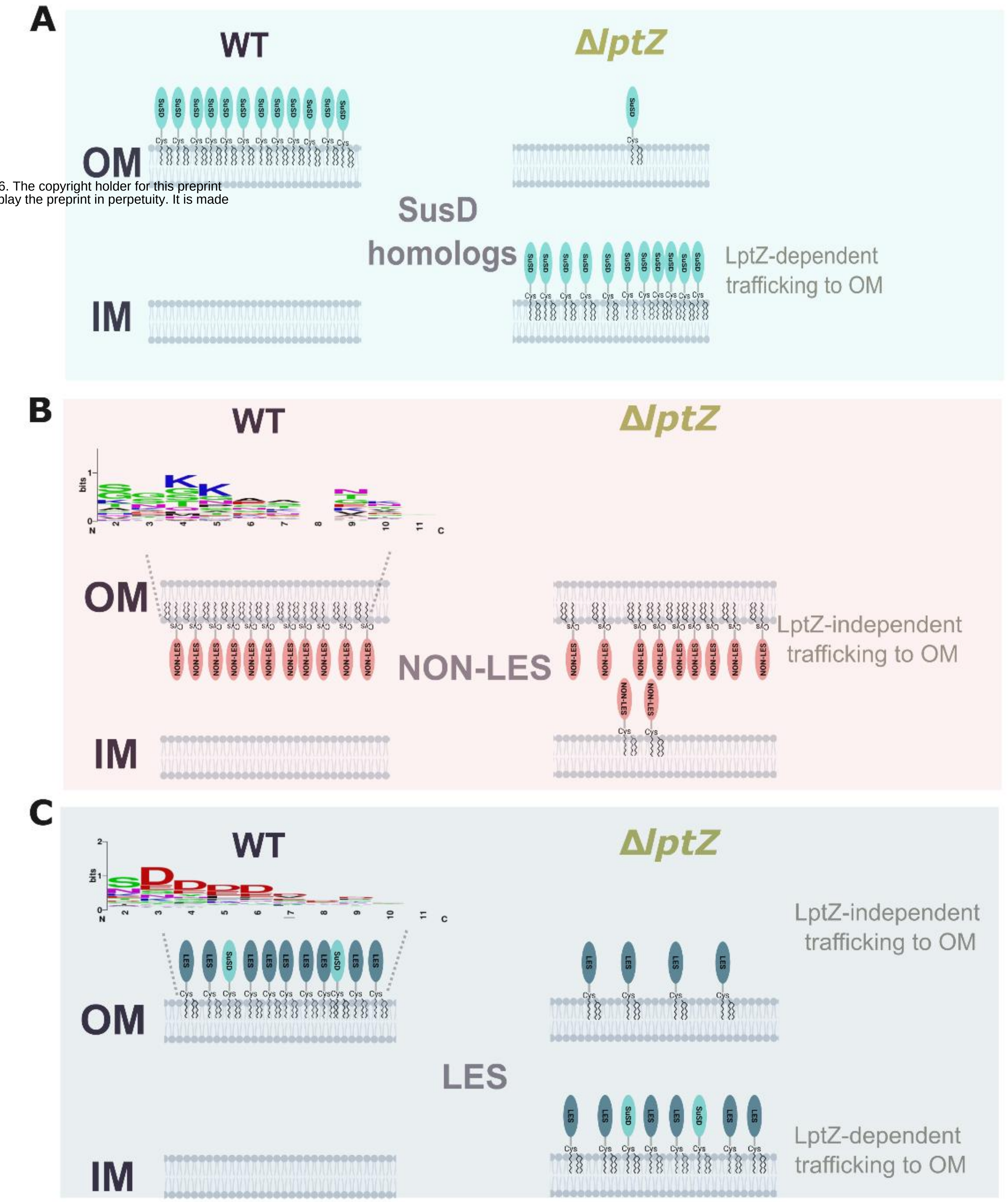

Figure S11: Schematic representation of lipoprotein transport mechanism, summarized from Figure 5. (A) In the WT strain, all SusD homologs localize to the outer surface of the OM, whereas in the Δ*lptZ* mutant, most SusD homologs are absent from the OM and retained in the IM as shown in Figure 5B. **(B)** Non-LES lipoproteins are largely unaffected in the Δ*lptZ* mutant and continue to localize to the periplasmic side of the OM, as shown in Figure 5D. **(C)** LES-containing lipoproteins, including SusD homologs, typically localize to the cell surface. In Δ*lptZ*, a subset accumulates in the IM and is absent from the cell surface, indicating impaired trafficking. In contrast, others still reach the surface, suggesting LptZ-independent transport for this group as shown in Figure 5E. Sequence logos representing the ten residues immediately downstream of the conserved lipoprotein attachment cysteine are shown for each group.

bioRxiv preprint doi: https://doi.org/10.64898/2026.10.02.756314; this version posted October 5, 2026. The copyright holder for this preprint (which was not certified by peer review) is the author/funder, who has granted bioRxiv a license to display the preprint in perpetuity. It is made available under a CC-BY 4.0 International license.

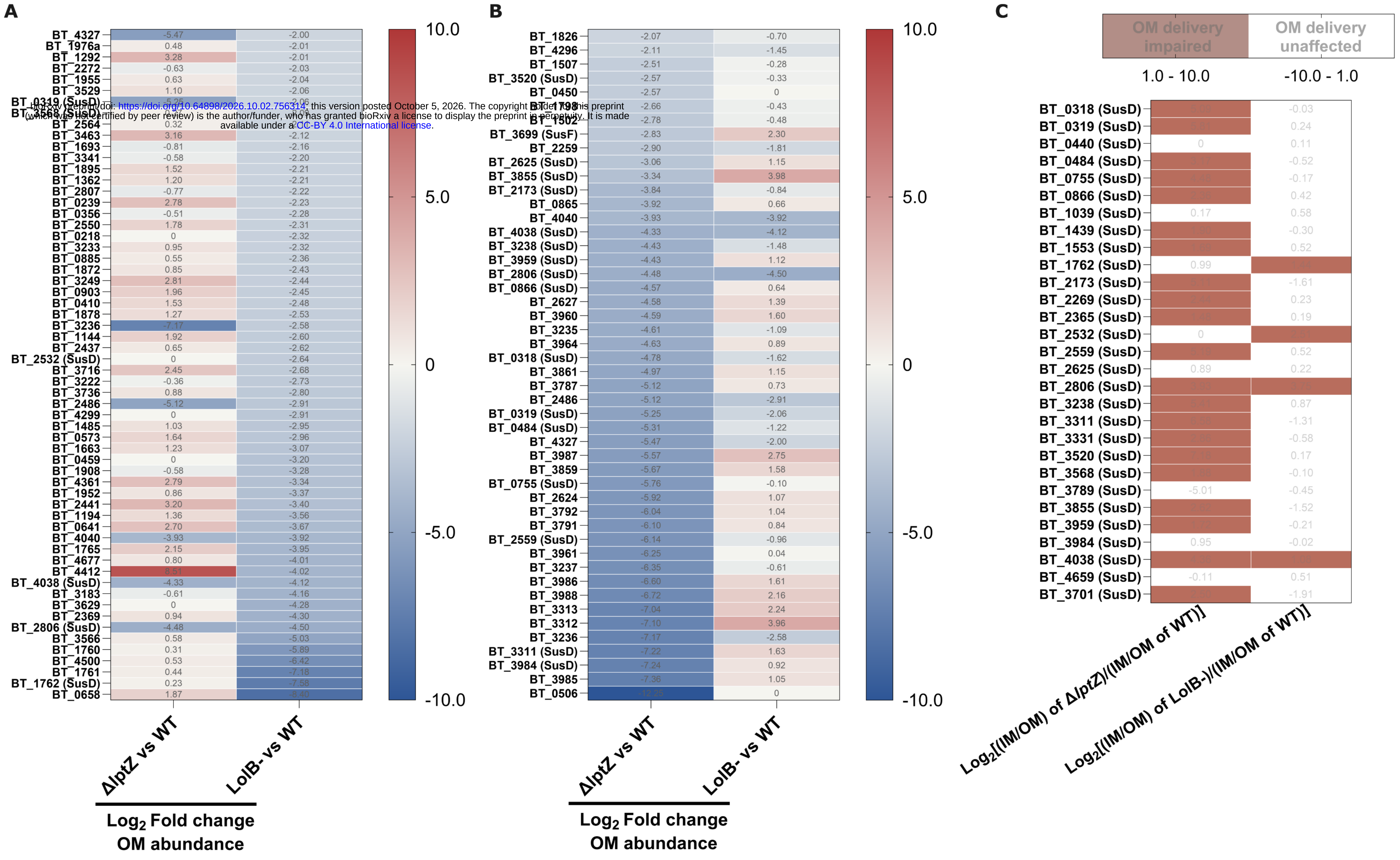

Figure S12: LptZ and LolB differentially affect OM lipoprotein trafficking. (A–B) Heat maps comparing changes in OM lipoprotein abundance in Δ*lptZ* and LolB- relative to WT. **(A)** Lipoproteins showing the strongest decreases in OM abundance in LolB- (log_2 fold change < −2) compared to WT are shown, with their corresponding changes in Δ*lptZ*. **(B)** Conversely, lipoproteins most strongly depleted from the OM in LolB- (log_2 fold change < −2) are shown, together with their corresponding changes in Δ*lptZ*. The largely distinct patterns of lipoprotein depletion indicate that the effects of Δ*lptZ* are not attributable to a general disruption of lipoprotein trafficking and differ from those caused by loss of LolB. **(C)** Comparison of SusD-like lipoprotein trafficking defects in ΔlptZ and LolB-. Relative changes in subcellular distribution were calculated as log_2[(IM/OM ratio in mutant)/(IM/OM ratio in WT)] and plotted as a heat map. Positive values (>1) indicate increased IM retention and impaired OM delivery, whereas values (<1) indicate no substantial trafficking defect. Most SusD-like lipoproteins show increased IM/OM ratios in Δ*lptZ* but not in LolB-, consistent with a specific requirement for LptZ in SusD-like lipoprotein delivery to the OM.

bioRxiv preprint doi: https://doi.org/10.64898/2026.10.02.756314; this version posted October 5, 2026. The copyright holder for this preprint (which was not certified by peer review) is the author/funder, who has granted bioRxiv a license to display the preprint in perpetuity. It is made available under a CC-BY 4.0 International license.

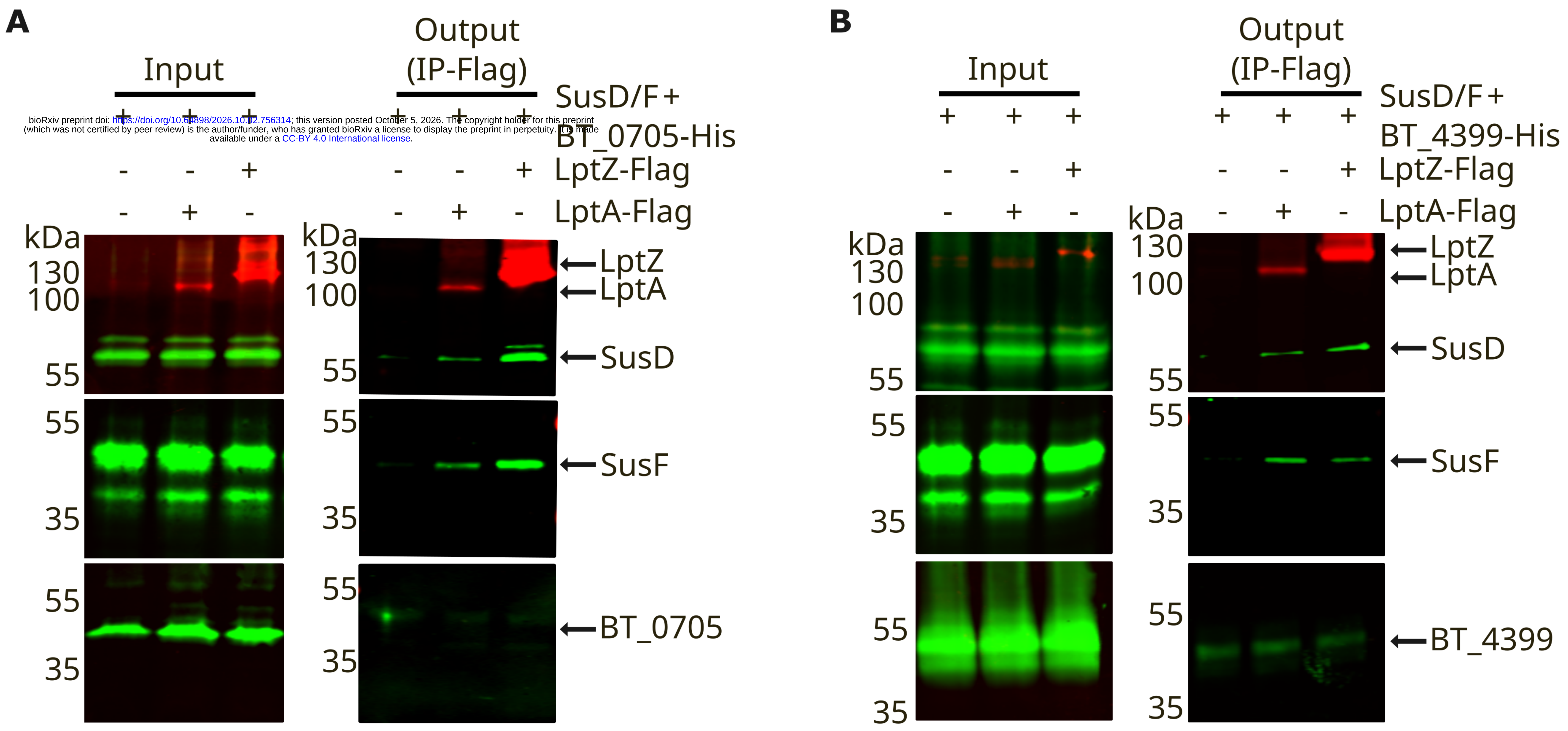

Figure S13: LES-containing lipoproteins selectively associate with the Lpt machinery. LptA and LptZ, each carrying a genomically encoded C-terminal 2xFLAG tag, were used as baits. **(A-B)** Maltose-induced SusD and SusF served as LES-containing substrates, whereas BT_0705 and BT_4399 represented non-LES outer-membrane lipoproteins and were constitutively expressed with C-terminal His tags. SusD and SusF were monitored with protein-specific antibodies, and BT_0705 and BT_4399 with an anti-His antibody. Both SusD and SusF were co-purified after DSP-mediated crosslinking with LptA and LptZ, whereas BT_0705 and BT_4399 were not relative to the untagged WT control. Experiments were independently repeated at least three times with similar results; one representative result is shown.

bioRxiv preprint doi: https://doi.org/10.64898/2026.10.02.756314; this version posted October 5, 2026. The copyright holder for this preprint (which was not certified by peer review) is the author/funder, who has granted bioRxiv a license to display the preprint in perpetuity. It is made available under a CC-BY 4.0 International license.

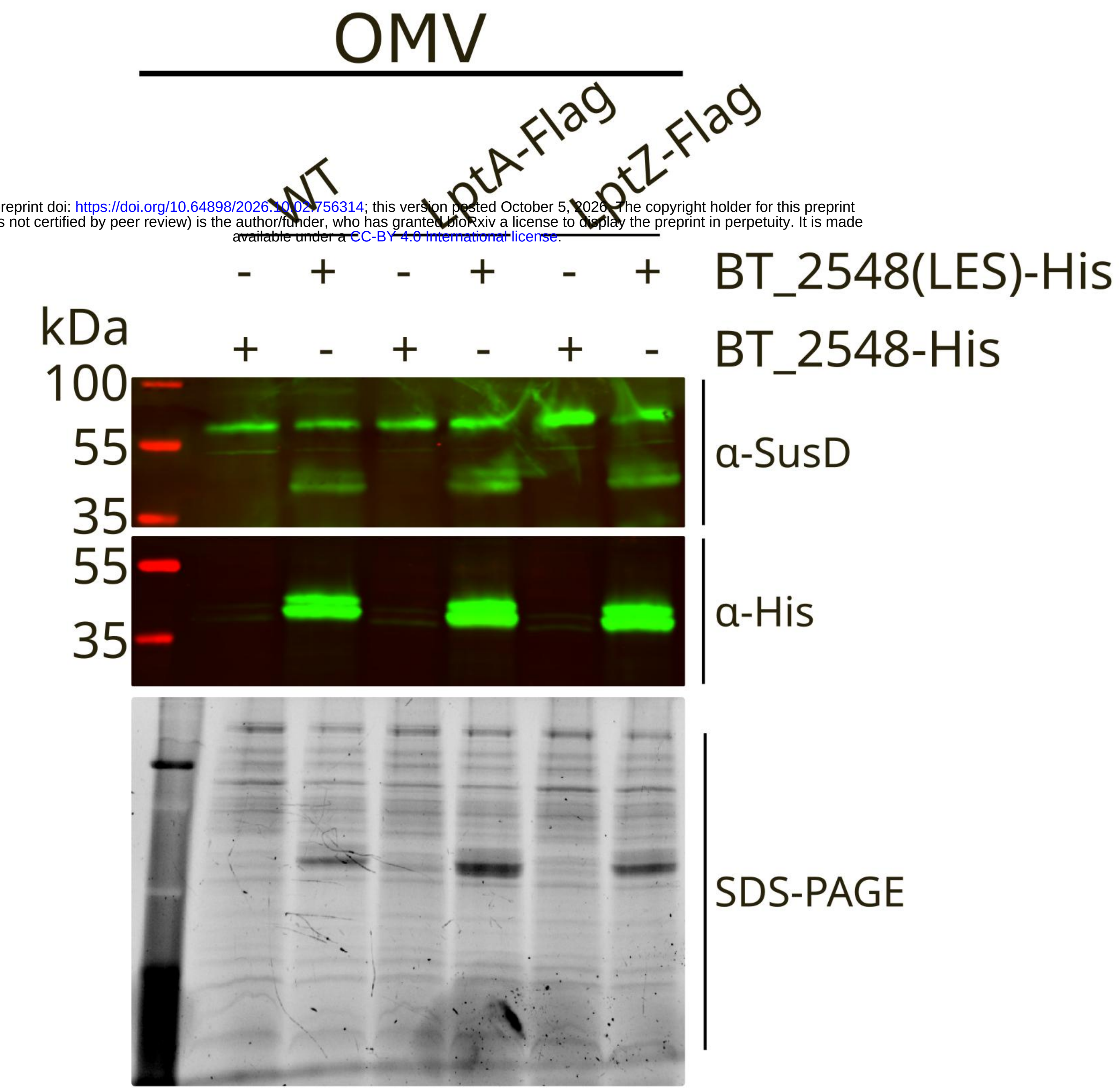

Figure S14: The SusD LES motif promotes incorporation of BT_2548 into OMVs. The native N-terminal non-LES sequence of BT_2548 was replaced with the LES sequence from SusD to generate BT_2548(LES). Both BT_2548 variants were C-terminally His-tagged. The resulting construct was introduced into WT and strains carrying genomically encoded C-terminal 2xFLAG tags on LptA or LptZ, and OMVs were isolated. **Upper panel:** Western blotting with an anti-SusD antibody confirmed comparable SusD abundance across strains. **Middle panel:** Immunoblotting with an anti-His antibody showed that replacement of the native non-LES with the SusD LES motif substantially increased the abundance of BT_2548 in the OMV fraction. **Lower panel:** SDS-PAGE analysis of the OMV fractions confirmed comparable OMV recovery across the strains. Experiments were independently performed at least twice with similar results; representative data are shown.

bioRxiv preprint doi: https://doi.org/10.64898/2026.10.02.756314; this version posted October 5, 2026. The copyright holder for this preprint (which was not certified by peer review) is the author/funder, who has granted bioRxiv a license to display the preprint in perpetuity. It is made available under a CC-BY 4.0 International license.

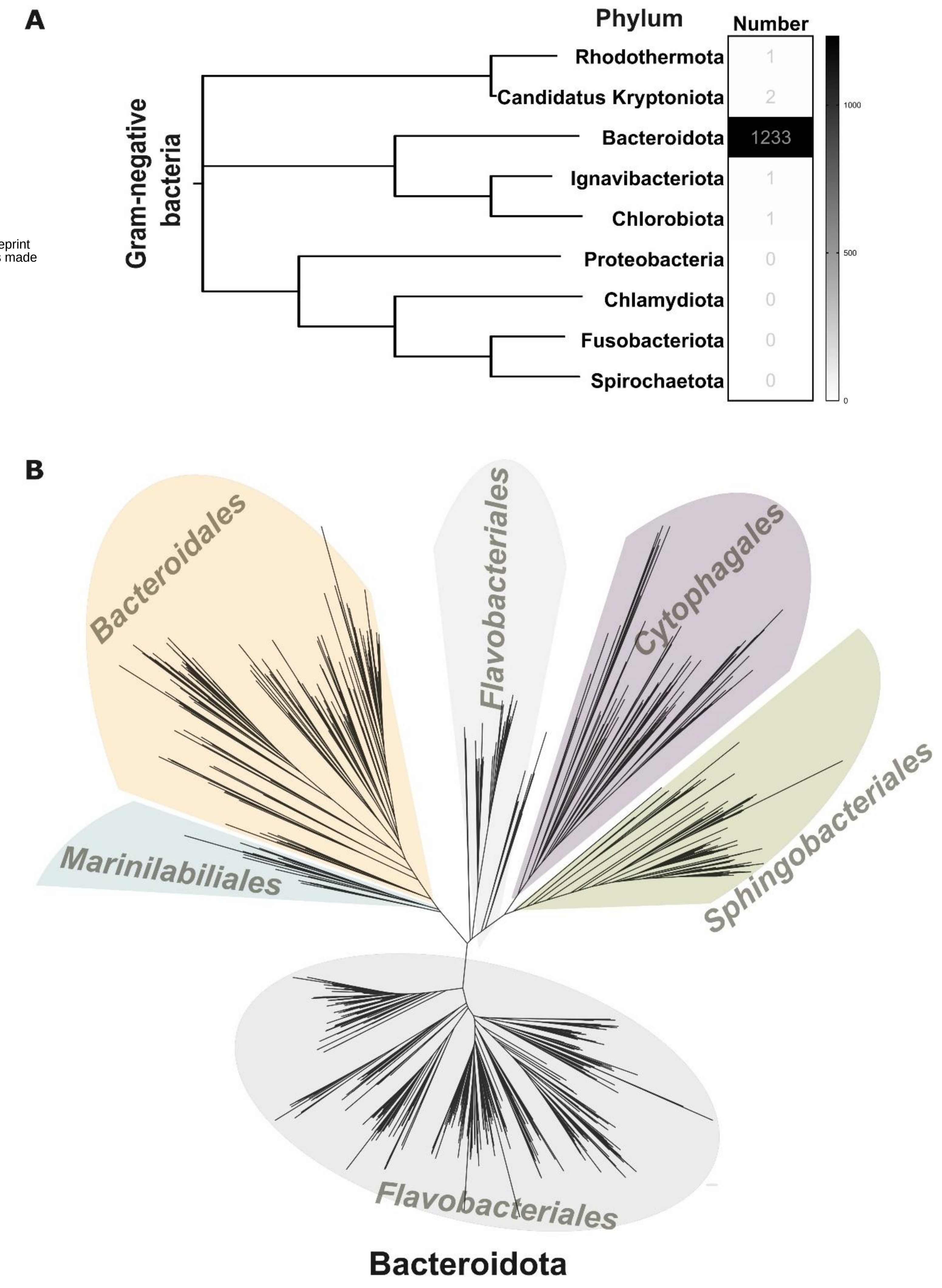

Figure S15: Functional conservation and phylogenetic distribution of LptZ. (**A**) Distribution of LptZ homologs across major phyla of Gram-negative bacteria. Numbers represent the count of LptZ homologs identified in each phylum during our analysis. **(B)** Phylogenetic tree of bacterial species encoding LptZ homologs. Branches are color-shaded by taxonomic order, including *Bacteroidales*, *Flavobacteriales*, *Cytophagales*, *Sphingobacteriales*, and *Marinilabiliales*.

bioRxiv preprint doi: https://doi.org/10.64898/2026.10.02.756314; this version posted October 5, 2026. The copyright holder for this preprint (which was not certified by peer review) is the author/funder, who has granted bioRxiv a license to display the preprint in perpetuity. It is made available under a CC-BY 4.0 International license.

Table S1: Top 50 abundant proteins in the OMV fraction of ΔLptZ versus WT

Gene names	Protein names	Predicted cellular localization	Log_2 Fold Change OMV (Δ*lptZ* vs WT)
BT_1998	Anaerobic ribonucleoside-triphosphate reductase	Cytosol	11.0921
BT_3876	30S ribosomal protein S9	Cytosol	10.8605
BT_0554	Glutamine--fructose-6-phosphate aminotransferase	Cytosol	10.8323
BT_2829	Arginine-tRNA ligase	Cytosol	10.7537
BT_4314	50S ribosomal protein L21	Cytosol	10.7233
BT_2713	30S ribosomal protein S8	Cytosol	10.475
BT_1499	DNA-binding protein HU	Cytosol	10.4695
BT_3877	30S ribosomal protein S2	Cytosol	10.4376
BT_3430	30S ribosomal protein S20	Cytosol	10.2642
BT_3995	Alanine--tRNA ligase	Cytosol	10.1802
BT_2714	30S ribosomal protein S14	Cytosol	10.157
BT_0578	Excinuclease ABC subunit A	Cytosol	10.1304
BT_3872	Pseudouridine synthase	Cytosol	10.0946
BT_4101	Alanine racemase	Cytosol	9.93964
BT_0425	50S ribosomal protein L20	Cytosol	9.89111
BT_1829	Chaperonin GroEL	Cytosol	9.87164
BT_3611	Glycine--tRNA ligase	Cytosol	9.8405
BT_2724	50S ribosomal protein L2	Cytosol	9.77836
BT_2710	30S ribosomal protein S5	Cytosol	9.75901
BT_1535	ABC transporter ATP-binding protein	Inner membrane	9.74155
BT_2701	DNA-directed RNA polymerase subunit alpha	Cytosol	9.68099
BT_4610	Protein RecA (Recombinase A)	Cytosol	9.61258
BT_1634	Putative aminoglycoside phosphotransferase	Not determined	9.51376
BT_2715	50S ribosomal protein L5	Cytosol	9.50339
BT_0408	YtxH domain-containing protein	membrane	9.4828
BT_0565	Putative heat shock protein		9.31365
BT_4362	Protein translocase subunit SecA	Inner membrane	9.30967
BT_0871	Diacylglycerol kinase-like, catalytic region	Cytosol	9.29115
BT_2416	GTP cyclohydrolase-2	Cytosol	9.24956
BT_0694	ABC transporter, ATP-binding protein	Inner membrane	9.24184
BT_4002	ATPase, ParA family	Cytosol	9.23574
BT_2703	30S ribosomal protein S11	Cytosol	9.23177
BT_4041	DUF349 domain-containing protein	Cytosol	9.21363

bioRxiv preprint doi: https://doi.org/10.64898/2026.10.02.756314; this version posted October 5, 2026. The copyright holder for this preprint (which was not certified by peer review) is the author/funder, who has granted bioRxiv a license to display the preprint in perpetuity. It is made available under a CC-BY 4.0 International license.

BT_2711	50S ribosomal protein L18	Cytosol	9.19412
BT_2552	Probable butyrate kinase (BK)	Cytosol	9.17372
BT_2721	30S ribosomal protein S3	Cytosol	9.13026
BT_1747	Pyruvate-flavodoxin oxidoreductase	Cytosol	9.095
BT_2720	50S ribosomal protein L16	Cytosol	9.03584
BT_0623	UDP-glucose 4-epimerase	Cytosol	9.00702
BT_0837	ATP-dependent protease	Cytosol	8.99484
BT_3214	S-adenosylmethionine:tRNA ribosyltransferase-isomerase	Cytosol	8.98287
BT_2717	50S ribosomal protein L14	Cytosol	8.97322
BT_3843	ATP-dependent Clp protease ATP-binding subunit ClpX	Cytosol	8.85366
BT_2704	30S ribosomal protein S13	Cytosol	8.80135
BT_3054	Succinate dehydrogenase flavoprotein subunit	Inner membrane	8.77293
BT_0557	carbamoyl-phosphate synthase	Cytosol	8.76847
BT_2734	DNA-directed RNA polymerase subunit beta	Cytosol	8.7472
BT_2787	50S ribosomal subunit assembly factor BipA	Cytosol	8.68427
BT_4372	Probable dual-specificity RNA methyltransferase RlmN	Cytosol	8.5796
BT_2162	30S ribosomal protein S18	Cytosol	8.53864

bioRxiv preprint doi: https://doi.org/10.64898/2026.10.02.756314; this version posted October 5, 2026. The copyright holder for this preprint (which was not certified by peer review) is the author/funder, who has granted bioRxiv a license to display the preprint in perpetuity. It is made available under a CC-BY 4.0 International license.

Table S2: Proteins co-purified with LptZ and LptA in the presence or absence of the crosslinker DSP, as shown in Fig. 3C- E. Lpt machinery components are indicated in green, LptZ in blue, and proteins co-purified with both LptA and LptZ in the presence of DSP in pink.

	Bait proteins (Conditions)			Predicted function
	LptZ (-DSP)	LptZ (+DSP)	LptA (+DSP)	
LptD (BT_2133)	✓	✓	✓	LPS transport
LptE (BT_4376)	✓	✓	✓	LPS transport
LptA (BT_3849)	✓	✓	✓	LPS transport
LptZ (BT_3341)	✓	✓	✓	Unknown
BT_0154	X	✓	✓	Unknown
BT_4000	X	✓	✓	Unknown
BT_4562	X	✓	✓	Unknown
BT_4563	X	✓	✓	Unknown
BT_0328	X	✓	✓	Unknown
BT_1085	X	✓	✓	Unknown

bioRxiv preprint doi: https://doi.org/10.64898/2026.10.02.756314; this version posted October 5, 2026. The copyright holder for this preprint (which was not certified by peer review) is the author/funder, who has granted bioRxiv a license to display the preprint in perpetuity. It is made available under a CC-BY 4.0 International license.

BT_0586	✓	X	X	Unknown
BT_0550	✓	X	X	Unknown
BT_2708	✓	X	X	Unknown
BT_1843	✓	X	X	Unknown
BT_4493	✓	X	X	Unknown
BT_1573	X	✓	X	Unknown
BT_0319	X	✓	X	Unknown
BT_2758	X	✓	X	Unknown
**LptF (BT_2417)**	**X**	**X**	**✓**	**LPS transport**
**LptG (BT_0834)**	**X**	**X**	**✓**	**LPS transport**
**LptC (BT_4369)**	**X**	**X**	**✓**	**LPS transport**
**LptB (BT_3837)**	**X**	**X**	**✓**	**LPS transport**
BT_0809	X	X	✓	Unknown
BT_2845	X	X	✓	Unknown
BT_0747	X	X	✓	Unknown

bioRxiv preprint doi: https://doi.org/10.64898/2026.10.02.756314; this version posted October 5, 2026. The copyright holder for this preprint (which was not certified by peer review) is the author/funder, who has granted bioRxiv a license to display the preprint in perpetuity. It is made available under a CC-BY 4.0 International license.

BT_3784	X	X	✓	Unknown
BT_3999	X	X	✓	Unknown

bioRxiv preprint doi: https://doi.org/10.64898/2026.10.02.756314; this version posted October 5, 2026. The copyright holder for this preprint (which was not certified by peer review) is the author/funder, who has granted bioRxiv a license to display the preprint in perpetuity. It is made available under a CC-BY 4.0 International license.

Table S3. A list of bacterial strains and plasmids used in this work.

**Strains used in this study**			
**Strain Name**	**Genotype**	**Comments**	**Source**
Bacteroides thetaiotaomicron VPI-5482	WT	Wild-type strain	Obtained from Jeffry I. Gordon laboratory
B. thetaiotaomicron VPI-5482 Δ*lptZ*	Δ*bt_3341*	*B. thetaiotaomicron* VPI-5482 strain with an in-frame deletion in the *lptZ* gene.	This study
B. thetaiotaomicron VPI-5482 Δ*lptZ*$_{comp}$	*B. thetaiotaomicron* VPI-5482 Δ*lptZ*:: pWW3452: LptZ	*B. thetaiotaomicron* VPI-5482 strain with a deletion in the *lptZ* gene, expressing LptZ fused to 3×FLAG and His_6 tags from the pWW3452 plasmid; used for complementation and membrane localization analyses	This study
B. thetaiotaomicron VPI-5482 pWW3452: BT_4362	*B. thetaiotaomicron* VPI-5482 :: pWW3452: BT_4362	*B. thetaiotaomicron* VPI-5482 strain expressing BT_4362 with FLAG (3X) and His_6 from pWW3452 plasmid ; used for membrane localization studies	This study
B. thetaiotaomicron VPI-5482 Δ*lptZ*:: pWW3515: LptZ	*B. thetaiotaomicron* VPI-5482 Δ*lptZ*:: pWW3515: LptZ	*B. thetaiotaomicron* VPI-5482 strain with a deletion in the *lptZ* gene, expressing LptZ fused to 3xFLAG and His_{10} tags from the pWW3515 plasmid; used for co-immunoprecipitation assays to identify LptZ-interacting partners.	This study
B. thetaiotaomicron VPI-5482 LptZ-2xFLAG	*B. thetaiotaomicron* VPI-5482 LptZ-2xFLAG	*B. thetaiotaomicron* VPI-5482 with genomically encoded C-terminally 2xFLAG-tagged LptZ	This study
B. thetaiotaomicron VPI-5482 LptA-2xFLAG	*B. thetaiotaomicron* VPI-5482 LptA-2xFLAG	*B. thetaiotaomicron* VPI-5482 with genomically encoded C-terminally 2xFLAG-tagged LptA	This study
B. thetaiotaomicron VPI-5482 LptZ-2xFLAG pWW3452: BT_0705-His10	*B. thetaiotaomicron* VPI-5482 LptZ-2xFLAG pWW3452: BT_0705-His10	*B. thetaiotaomicron* VPI-5482 with genomically encoded C-terminally 2xFLAG-tagged LptZ expressing BT_0705 fused to His_{10} tags from the pWW3452 plasmid; used for co-immunoprecipitation .	This study
B. thetaiotaomicron VPI-5482 LptZ-2xFLAG pWW3452: BT_2548-His10	*B. thetaiotaomicron* VPI-5482 LptZ-2xFLAG pWW3452: BT_2548-His10	*B. thetaiotaomicron* VPI-5482 with genomically encoded C-terminally 2xFLAG-tagged LptZ expressing BT_2548 fused to His_{10} tags from the pWW3452	This study

bioRxiv preprint doi: https://doi.org/10.64898/2026.10.02.756314; this version posted October 5, 2026. The copyright holder for this preprint (which was not certified by peer review) is the author/funder, who has granted bioRxiv a license to display the preprint in perpetuity. It is made available under a CC-BY 4.0 International license.

		plasmid; used for co-immunoprecipitation.	
B. thetaiotaomicron VPI-5482 LptZ-2xFLAG pWW3452: BT_4399-His10	*B. thetaiotaomicron* VPI-5482 LptZ-2xFLAG pWW3452: BT_4399-His10	*B. thetaiotaomicron* VPI-5482 with genomically encoded C-terminally 2xFLAG-tagged LptZ expressing BT_4399 fused to His_{10} tags from the pWW3452 plasmid; used for co-immunoprecipitation.	This study
B. thetaiotaomicron VPI-5482 LptZ-2xFLAG pWW3452: BT_2548 (LES)-His10	*B. thetaiotaomicron* VPI-5482 LptZ-2xFLAG pWW3452: BT_2548-His10	*B. thetaiotaomicron* VPI-5482 with genomically encoded C-terminally 2×FLAG-tagged LptZ, expressing BT_2548 in which the N-terminal region was replaced with the N-terminal signal sequence and LES domain of SusD and fused to a His_{10} tag from plasmid pWW3452; used for co-immunoprecipitation.	This study
B. thetaiotaomicron VPI-5482 LptA-2xFLAG pWW3452: BT_0705-His10	*B. thetaiotaomicron* VPI-5482 LptA-2xFLAG pWW3452: BT_0705-His10	*B. thetaiotaomicron* VPI-5482 with genomically encoded C-terminally 2xFLAG-tagged LptA expressing BT_0705 fused to His_{10} tags from the pWW3452 plasmid; used for co-immunoprecipitation .	This study
B. thetaiotaomicron VPI-5482 LptA-2xFLAG pWW3452: BT_2548-His10	*B. thetaiotaomicron* VPI-5482 LptA-2xFLAG pWW3452: BT_2548-His10	*B. thetaiotaomicron* VPI-5482 with genomically encoded C-terminally 2xFLAG-tagged LptA expressing BT_2548 fused to His_{10} tags from the pWW3452 plasmid; used for co-immunoprecipitation.	This study
B. thetaiotaomicron VPI-5482 LptA-2xFLAG pWW3452: BT_4399-His10	*B. thetaiotaomicron* VPI-5482 LptA-2xFLAG pWW3452: BT_4399-His10	*B. thetaiotaomicron* VPI-5482 with genomically encoded C-terminally 2xFLAG-tagged LptA expressing BT_4399 fused to His_{10} tags from the pWW3452 plasmid; used for co-immunoprecipitation.	This study
B. thetaiotaomicron VPI-5482 LptA-2xFLAG pWW3452: BT_2548 (LES)-His10	*B. thetaiotaomicron* VPI-5482 LptA-2xFLAG pWW3452: BT_2548-His10	*B. thetaiotaomicron* VPI-5482 with genomically encoded C-terminally 2x FLAG-tagged LptA, expressing BT_2548 in which the N-terminal region was replaced with the N-terminal signal sequence and LES domain of SusD and fused to a His_{10} tag from plasmid	This study

bioRxiv preprint doi: https://doi.org/10.64898/2026.10.02.756314; this version posted October 5, 2026. The copyright holder for this preprint (which was not certified by peer review) is the author/funder, who has granted bioRxiv a license to display the preprint in perpetuity. It is made available under a CC-BY 4.0 International license.

		pWW3452; used for co-immunoprecipitation.	
Escherichia coli	S17-1λ*pir*	Conjugation donor strain to introduce plasmids in *B. thetaiotaomicron*	Obtained from Jeffry I. Gordon laboratory
Escherichia coli	BL21(DE3)	Expression of proteins to check protein-protein interaction	Obtained from Tracey Raivio's laboratory
**Plasmids used in this study**			
**Name**	**Description**	**Purpose**	**Reference/ Source**
pS1E1	a Amp^R and gentamicin^R containing suicide vector	Used to generate deletions in *Bt* genomes	[1]
pS1E1:BT_3341	The pS1E1 plasmid contains a 750 bp upstream-downstream flanking sequence of the *bt_3341* gene.	Used to generate deletions *bt_3341/lptZ* gene in *Bt* genomes	This study
pS1E1:BT_3341-2xFLAG	The pS1E1 plasmid contains 1,000-bp flanking regions surrounding the 2xFLAG insertion site of BT_3341(LptZ) and the 2xFLAG-encoding sequence.	Used to generate deletions *bt_3341/lptZ* -2xFLAG in *Bt* genomes	This study
pS1E1:BT_3849 (LptA)-2xFLAG	The pS1E1 plasmid contains 1,000-bp flanking regions surrounding the 2xFLAG insertion site of BT_3341(LptZ) and the 2xFLAG-encoding sequence.	Used to generate deletions *bt_3849/lptA* -2xFLAG in *Bt* genomes	This study
pWW3452	An Amp^R and Erm^R plasmid carrying 3xFLAG and His_6	Use for constitutive expression of proteins in *Bt*	[2]
pWW3515	An Amp^R and Erm^R plasmid carrying for constitutive expression of proteins in *Bt*	Use for constitutive expression of proteins in *Bt*	[2]

bioRxiv preprint doi: https://doi.org/10.64898/2026.10.02.756314; this version posted October 5, 2026. The copyright holder for this preprint (which was not certified by peer review) is the author/funder, who has granted bioRxiv a license to display the preprint in perpetuity. It is made available under a CC-BY 4.0 International license.

pWW3452:LptZ	pWW3452 plasmid encoding LptZ fused to a C-terminal 3xFLAG and His_6	Used for constitutive expression of LptZ protein in *Bt* to complement the Δ*lptZ* mutant	This study
pWW3515:LptZ	pWW3515 plasmid encoding LptZ fused to a C-terminal 3xFLAG and His_{10}	Used for constitutive expression of proteins in *Bt* for pull-down experiments to identify LptZ interaction partners	This study
pWW3452:BT_4362	pWW3452 plasmid encoding BT_4362 fused to a C-terminal 3xFLAG and His_6	Used for constitutive expression of BT_4362 protein in *Bt*	This study
pWW3452:BT_0705	pWW3452 plasmid encoding BT_0705 fused to a C-terminal His_{10}	Used for constitutive expression of BT_0705 protein in *Bt*	This study
pWW3452:BT_2548	pWW3452 plasmid encoding BT_2548 fused to a C-terminal His_{10}	Used for constitutive expression of BT_2548 protein in *Bt*	This study
pWW3452:BT_4399	pWW3452 plasmid encoding BT_4399 fused to a C-terminal His_{10}	Used for constitutive expression of BT_4399 protein in *Bt*	This study
pWW3452:BT_2548 (LES)	pWW3452 plasmid: BT_2548 in which the N-terminal region was replaced with the N-terminal signal sequence and LES domain of SusD and fused to a His_{10} tag	Used for constitutive expression of BT_2548 (LES) protein in *Bt*	This study
pBAD/Myc-His	An Amp^R vector with P*bad* promoter	Used for arabinose-inducible expression of proteins	invitrogen
pBAD33.1	A Cm^R vector with P*bad* promoter	Used for arabinose-inducible expression of proteins	[3]
pBAD33.1:LptZ	pBAD33.1 carrying the CDS of *bt_3341* fused to a C-terminal 1xFLAG tag	Used for arabinose-inducible expression in *E. coli* to assess protein-protein interactions via pull-down assays	This study
pBAD/Myc-His:LptD	pBAD/Myc-His carrying the CDS of *bt_2133* fused to a C-terminal His_{10} tag	Used for arabinose-inducible expression in *E. coli* to assess protein-protein interactions via pull-down assays	This study
pBAD/Myc-His:LptE	pBAD/Myc-His carrying the CDS of *bt_4376* fused to a	Used for arabinose-inducible expression in *E. coli* to assess protein-protein interactions via	This study

bioRxiv preprint doi: https://doi.org/10.64898/2026.10.02.756314; this version posted October 5, 2026. The copyright holder for this preprint (which was not certified by peer review) is the author/funder, who has granted bioRxiv a license to display the preprint in perpetuity. It is made available under a CC-BY 4.0 International license.

	C-terminal His_{10} tag	pull-down assays	
pBAD/Myc-His:LptA	pBAD/Myc-His carrying the CDS of *bt_3849* fused to a C-terminal His_{10} tag	Used for arabinose-inducible expression in *E. coli* to assess protein-protein interactions via pull-down assays	This study
pBAD/Myc-His:LptC	pBAD/Myc-His carrying the CDS of *bt_4369* fused to a C-terminal His_{10} tag	Used for arabinose-inducible expression in *E. coli* to assess protein-protein interactions via pull-down assays	This study
pBAD/Myc-His:LptG	pBAD/Myc-His carrying the CDS of *bt_0834* fused to a C-terminal His_{10} tag	Used for arabinose-inducible expression in *E. coli* to assess protein-protein interactions via pull-down assays	This study
pBAD/Myc-His:LptF	pBAD/Myc-His carrying the CDS of *bt_2133* fused to a C-terminal His_{10} tag	Used for arabinose-inducible expression in *E. coli* to assess protein-protein interactions via pull-down assays	This study
pBAD/Myc-His:LptB	pBAD/Myc-His carrying the CDS of *bt_3837* fused to a C-terminal His_{10} tag	Used for arabinose-inducible expression in *E. coli* to assess protein-protein interactions via pull-down assays	This study
pKNT25	Carrying T25 domain of *Bordetella pertussis* adenylate cyclase	For bacterial two-hybrid assay	[4]
pUT18	Carrying T18 domain, of *Bordetella pertussis* adenylate cyclase	For the bacterial two-hybrid assay	[4]
pUT18: LptZ	pUT18 carrying the CDS of *bt_3341* fused to a C-terminal T18 domain	For the bacterial two-hybrid assay	This study
pKNT25: LptD	pKNT25 carrying the CDS of *bt_2133* fused to a C-terminal T25 domain	For the bacterial two-hybrid assay	This study
pKNT25: LptE	pKNT25 carrying the CDS of *bt_4376* fused to a C-terminal T25 domain	For the bacterial two-hybrid assay	This study
pKNT25: LptA	pKNT25 carrying the CDS of *bt_3849* fused to a C-terminal T25 domain	For the bacterial two-hybrid assay	This study
pKNT25: LptC	pKNT25 carrying the CDS of *bt_4369* fused to a C-terminal T25 domain	For the bacterial two-hybrid assay	This study
pKNT25: LptG	pKNT25 carrying	For the bacterial two-hybrid	This study

bioRxiv preprint doi: https://doi.org/10.64898/2026.10.02.756314; this version posted October 5, 2026. The copyright holder for this preprint (which was not certified by peer review) is the author/funder, who has granted bioRxiv a license to display the preprint in perpetuity. It is made available under a CC-BY 4.0 International license.

	the CDS of *bt_0834* fused to a C-terminal T25 domain	assay	
pKNT25: LptF	pKNT25 carrying the CDS of *bt_2417* fused to a C-terminal T25 domain	For the bacterial two-hybrid assay	This study
pKNT25: LptB	pKNT25 carrying the CDS of *bt_3837* fused to a C-terminal T25 domain	For the bacterial two-hybrid assay	This study

References:

1. Bencivenga-Barry, N.A., Lim, B., Herrera, C.M., Trent, M.S., and Goodman, A.L. (2020). Genetic Manipulation of Wild Human Gut *Bacteroides*. J. Bacteriol. *202*. https://doi.org/10.1128/JB.00544-19.

2. Whitaker, W.R., Shepherd, E.S., and Sonnenburg, J.L. (2017). Tunable Expression Tools Enable Single-Cell Strain Distinction in the Gut Microbiome. Cell *169*, 538-546.e12. https://doi.org/10.1016/j.cell.2017.03.041.

3. Chung, H.S., and Raetz, C.R.H. (2010). Interchangeable Domains in the Kdo Transferases of *Escherichia coli* and *Haemophilus influenzae*. Biochemistry *49*, 4126–4137. https://doi.org/10.1021/bi100343e.

4. Karimova, G., Ullmann, A., and Ladant, D. (2001). Protein-protein interaction between Bacillus stearothermophilus tyrosyl-tRNA synthetase subdomains revealed by a bacterial two-hybrid system. J. Mol. Microbiol. Biotechnol. *3*, 73–82.
